# The Impact of Leader Proactivity on Follower Proactivity: A Chain Mediation Model

**DOI:** 10.3389/fpsyg.2022.781110

**Published:** 2022-03-22

**Authors:** Kaixin Zhang, Zilong Cui

**Affiliations:** ^1^International School of Business and Management, Shanghai International Studies University, Shanghai, China; ^2^Yatai College of Business Administration, Jilin University of Finance and Economics, Changchun, China

**Keywords:** leader, proactivity, value congruence, role breadth self-efficacy, felt responsibility for constructive change

## Abstract

This study aims to explore the linking mechanisms underlying the relationship between leader proactivity and follower proactivity. Drawing on social learning theory, the present research investigates the effects of leader proactivity on follower proactivity by developing a chain mediation model. An analysis of three-wave lagged data (*N* = 575) on 575 employees of six firms in China shows that leader proactivity is positively related to follower proactivity and that employees’ role breadth self-efficacy (RBSE) and felt responsibility for constructive change (FRCC) mediate this relationship. The analytical results also show that leader proactivity facilitates follower value congruence, which in turn enhances followers’ RBSE and FRCC and ultimately promotes followers’ proactivity. The results extend the current proactivity literature and fill the research gap by investigating the relationship between leader proactivity and follower proactivity. The current study also contributes to the literature by identifying the mediating mechanism of the “can do” and “reason to” mechanisms that link leader proactivity to follower proactivity.

## Introduction

With increasing uncertainty in work environments and in customer needs and expectations, employees’ proactivity not only provides the organization with a successful service but also offers the organization a significant competitive advantage (Thomas et al., 2010; Raub and Liao, 2012; Horng et al., 2016). Proactive behaviors involve individuals making self-directed and future-oriented changes in their actions in their environment or work role (Griffin et al., 2007; Parker et al., 2010). Employee proactivity can take many forms, such as taking charge (Morrison and Phelps, 1999), expressing their voice (Morrison, 2011), taking personal initiative (Fay and Frese, 2001), seeking feedback (Ashford and Cummings, 1985; Anseel et al., 2015), taking initiative in their career (Seibert et al., 2001), and crafting their job (Wrzesniewski and Dutton, 2001; Tims et al., 2012). In the last 20 years, proactive behavior has been highly prized in organizations and by managers for its potential to benefit organizations, such as by enhancing organizational and individual performance and changing the long-term working environment (Tornau and Frese, 2013). Proactivity research to date, however, has focused on how to motivate employees to engage in proactive behavior and confirming several different personal characteristics and job characteristics as antecedents (Parker et al., 2006, 2010). Additionally, the existing literature has yielded some knowledge regarding employee-level proactivity, but it has focused less on manager or leader proactivity. Previous studies suggest that manager proactivity can motivate frontline employees and increase unit-level collective performance (Crossley et al., 2013). Due to leaders’ power and prominence in the organizational hierarchy, leaders are considered critical catalysts and have a remarkable influence on motivating subordinates’ initiative. For example, leadership (e.g., transformational, authentic, spiritual, servant, ethical, and empowering leadership) has been found to be positively associated with subordinates’ proactive behavior (Den Hartog and Belschak, 2012; Cheng et al., 2014; Hu et al., 2018; Kim and Beehr, 2018; Weiss et al., 2018; Chen et al., 2019; Mostafa and El-Motalib, 2019). The quality of the relationship between leaders and employees (also known as leader-member exchange, or LMX) has been shown to be positively related to subordinates’ proactive behavior (e.g., innovation and creativity, voice, and change-oriented organizational citizenship behaviors) (Tierney et al., 1999; Van Dyne et al., 2002; Bettencourt, 2004; Janssen and Van Yperen, 2004; Van Dyne et al., 2008; Botero and Van Dyne, 2009). However, there is little information on the relationship between leaders’ proactivity and followers’ proactivity. Generally, leaders’ behavior can influence their subordinates’ behavior through trickle-down effects (Wayne et al., 2008). Can leaders’ proactivity fuel followers’ proactivity? If so, what is the internal mechanism of this effect?

**FIGURE 1:**
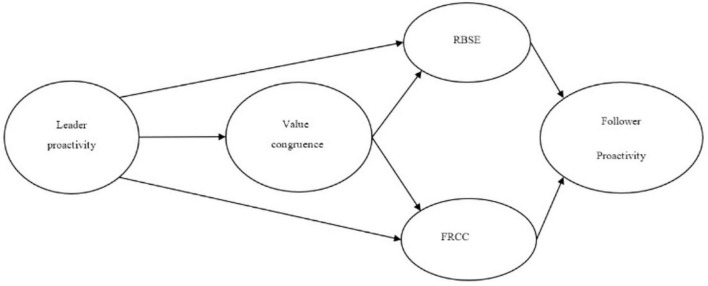
Conceptual model.

To fill this research gap, drawing on social information processing theory and social learning theory, we develop a trickle-down process model to investigate the relationship between leaders’ proactivity and followers’ proactivity. Social information processing theory suggests that the emotion, cognition, attitude, and behavior of leaders are important information in work situations, and followers use this information as clues to construct and interpret events and then react accordingly (Salancik and Pfeffer, 1978). Possessing power in an organization, leaders tailor values for followers to espouse and reward followers by how much the leader focuses on the organization’s interests (Stern et al., 1999). Proactive leaders set challenging goals for followers, satisfy followers’ social needs and build interpersonal relations with followers, causing followers to identify with leaders’ proactive behavior (Crossley et al., 2013; Huang et al., 2020). Thus, followers are more likely to identify with and learn values from proactive leaders, which ultimately facilitates value congruence between leaders and followers (Larson et al., 1986; Lam et al., 2018; Peng et al., 2020). Following social learning theory, we first suggest that value congruency among followers and proactive leaders enhances followers’ self-efficacy in performing a wide range of job tasks (role−breadth self−efficacy, RBSE) through role modeling (Lee et al., 2017). Furthermore, we argue that value similarity between proactive leaders and followers makes followers take on more responsibility (felt responsibility for constructive change, FRCC) to be proactive like their leaders (Marstand et al., 2018). Based on the proactive motivation model, RBSE and FRCC are “can do” and “reason to” motivational states that inspire individual proactivity. Taken together, we propose that the effect of leader proactive behavior on follower proactive behavior trickles down through our proposed chain. Additionally, we propose that leader proactive behavior is associated with value congruence, which corresponds with RBSE and ultimately increases follower proactivity. We also postulate that leader proactive behavior is associated with value congruence, which corresponds with FRCC and ultimately increases follower proactivity. The theoretical model is presented in Figure 1.

Most literature on proactive behavior focuses on the employee perspective and devotes little attention to leader or manager proactivity. Our model contributes to the proactivity literature in three ways. First, existing studies have focused on how leader-follower congruence in a proactive personality influences followers’ attitudes and behaviors (Lam et al., 2018; Huang et al., 2020; Peng et al., 2020). We extend the current proactivity literature and fill the research gap by investigating the relationship between leader proactivity and follower proactivity. Second, by examining RBSE and FRCC as mediators of the relationship between leader proactivity and follower proactivity, our work expands the theoretical understanding of proactivity by identifying and testing both the “can do” and “reason to” mechanisms that link leader proactivity to follower proactivity. Third, existing studies have paid little attention to the underlying mechanisms through which leaders’ proactivity trickles down to followers’ proactivity. Based on social learning theory, we develop and test a trickle-down model in which leaders’ proactivity influences followers’ proactivity *via* a social learning process.

## Theory and Hypotheses

### Leader Proactivity and Follower Proactivity

Existing research shows that leaders play an important role in motivating followers to engage in various forms of proactive behavior (Axtell et al., 2000; Madjar et al., 2002; Gao et al., 2011). Leaders can be seen as key catalysts in forming employees’ perceptions of how their organization supports them and how they are expected to navigate the work environment (Naumann and Bennett, 2000). Because of their power within an organization, leaders allocate resources and opportunities and reward and punish employees in the interest of the organization based on their values. Based on social learning theory, employees are inclined to emulate the behaviors of their role models, such as leaders and coworkers (Bommer et al., 2003; Ehrhart and Naumann, 2005; Mayer et al., 2009). Previous studies suggest that leaders may influence the behaviors of followers by role modeling, leading followers to emulate leaders’ behavior (Jaussi and Dionne, 2003; Palanski and Yammarino, 2011; Yaffe and Kark, 2011). Proactive leaders are attractive to employees. For example, Deluga (1998) found that American presidents who were rated as having a more proactive personality were also rated as displaying more charismatic leadership. When leaders exhibit proactive behavior, they set challenging goals by envisioning a desirable future, taking charge to improve work efficiency, and providing subordinate information and help (Crossley et al., 2013; Rashkovits, 2019). Under proactive leaders, subordinates have strong motivation to learn and imitate leaders’ values, cognitive patterns, and behaviors. Thus, leaders who display high proactivity are likely to develop followers who also display high proactivity. Xin et al. (2020) proposed that subordinates imitate the job crafting of their leaders. Hence, we hypothesize that:

Hypothesis 1: Leader proactivity is positively related to follower proactivity.

### The Mediating Role of Role Breadth Self-Efficacy Between Leader Proactivity and Follower Proactivity

Role breadth self-efficacy is a form of self-efficacy that means a person’s self-perceived ability to implement broader tasks in addition to immediate technical work (Parker, 2000). RBSE has been demonstrated to have positive effects on innovative behavior (Odoardi, 2015), change-oriented organizational citizenship behavior (López-Domínguez et al., 2013), perceived employability (Kim et al., 2015), and proactive behavior (Ohly and Fritz, 2007; Hwang et al., 2015). We expect a positive effect of leader proactivity on followers’ RBSE. Proactive leaders are regarded as having strong leader visions that are future-focused and change-oriented and as striving to change their environment (Wu and Wang, 2011). According to social learning theory, employees can shape and develop their self-efficacy beliefs by learning, acquiring experience and persuasion. Proactive leaders set challenging goals for followers, encourage followers to take on responsibilities, and provide followers guidance (Crossley et al., 2013; Lam et al., 2018; Rashkovits, 2019). Thus, proactive leaders may inspire followers to go beyond role expectations and complete tasks on their own. Followers become more inclined to develop their self-efficacy by performing various tasks and accumulating mastery experience. Moreover, proactive leaders go beyond focusing on core tasks, implement changes by creating a challenging vision and maintain the motivation of their subordinates, thus facilitating high self-efficacy in their subordinates (Wu and Wang, 2011). These behaviors can also facilitate followers’ self-efficacy through role modeling so that employees can learn from their leaders’ experiences.

We expect that RBSE is positively related to proactive behavior. Proactive behavior is risky because it challenges the current *status quo*. Proactive behavior is not always welcomed by leaders and coworkers (Parker et al., 2019). Thus, when people are proactive, they should feel that they are capable of behaving proactively. This means that when individuals have high self-efficacy or self-perceived ability for proactive action, they are more likely to cope with setbacks and take proactive action. RBSE depicts individuals’ self-perceived ability to engage in proactivity beyond their job requirements (Parker, 1998), and there is strong evidence that RBSE can motivate people to engage in various types of proactivity (Parker, 2000; Ohly and Fritz, 2007; Fuller et al., 2012; Hwang et al., 2015).

We propose that leader proactivity has indirect effects on follower proactivity through RBSE. Regarding the proactive motivation model proposed by Parker et al. (2010), proactive leaders inspire followers to go beyond role expectations, encourage change, and provide a clear vision. These behaviors provide “can do” motivation, which ultimately increases followers’ proactive behavior. In previous studies, RBSE has been found to mediate the relationship between leader factors and follower proactivity (Detert and Burris, 2007; Den Hartog and Belschak, 2012; López-Domínguez et al., 2013; Li et al., 2015). Therefore, we hypothesize the following:

Hypothesis 2: Role breadth self-efficacy mediates the relationship between leader proactivity and follower proactivity.

### The Mediating Role of Felt Responsibility for Constructive Change Leader Proactivity and Follower Proactivity

Felt responsibility for constructive change refers to a flexible mental state in which employees experience a willingness and responsibility for continually redefining performance (Fuller et al., 2006). When people perceive the duty to make changes, they want to improve organizational processes, develop new work procedures and correct problems in organizations. According to social learning theory, leaders’ proactive behavior leads to changes in the environment and is associated with personal loss and risk, indicating leaders’ high commitment to their organization’s goals and missions. Proactive leaders are regarded as perseverant and tough-minded when facing possible setbacks (Wu and Wang, 2011). Therefore, proactive leaders can strengthen employees’ perception of responsibility through role modeling. Meanwhile, leaders are important organizational agents for employees (Pan et al., 2012). Proactive leaders think and take action in advance, and they may be more inclined than non-proactive leaders to pay attention to the needs and difficulties of employees. Rashkovits (2019) argues that a highly proactive nurse leader can attenuate the adverse effect of nursing team workload. In line with social exchange theory (Blau, 1964), when employees have a positive perception of proactive leaders, they feel a sense of responsibility for the organization following the principle of reciprocity. Therefore, by extension, it seems likely that leader proactivity enhances employees’ felt responsibility for constructive change.

When people feel responsible for constructive change, they are more inclined to reflect a proactive conceptualization of their obligations at work (Fuller et al., 2006). This means that the FRCC is a proactive mindset that encourages people to engage in proactivity for organizational functional change. Parker et al. (2010) suggest that FRCC is a kind of proactive motivation that facilitates personal initiative. Previous research has found much empirical evidence on the positive effect of FRCC on proactive behavior (Fuller et al., 2012). For instance, Fuller et al. (2006) suggest that FRCC has a positive correlation with employee voice (i.e., constructive, change-oriented communication). Similarly, Arain et al. (2019) propose that the FRCC predicts employees’ prohibitive voice.

We expect that FRCC mediates the relationship between leader proactivity and follower proactivity. Regarding the proactive motivation perspective proposed by Parker et al. (2010), the FRCC can be seen as a “reason to” motivational state that interprets the link between leader factors and follower proactivity (López-Domínguez et al., 2013; Arain et al., 2019). This means that leaders provide a reason for followers to engage in proactivity. Based on the above analysis, proactive leaders can strengthen employees’ perception of responsibility for constructive change through role modeling, which facilitates followers’ proactive behavior. Therefore, we hypothesize the following:

Hypothesis 3: Felt responsibility for constructive change mediates the relationship between leader proactivity and follower proactivity.

### The Chain Mediating Role of Value Congruence, Role Breadth Self-Efficacy, and Felt Responsibility for Constructive Change

As essential organizational agents, leaders transform the values of their followers into collective values that followers can share (Jung and Avolio, 2000). We suggest that leader proactive behavior is positively related to value congruence between leaders and followers. Proactive leaders seek to envision future challenges, search for relevant information, and set relevant goals (Wu and Wang, 2011). According to Kirkpatrick and Locke (1996), leaders with a high-quality vision of the future promote value congruence between leaders and followers. Furthermore, proactive leaders can (a) offer explicit directions for proper conduct, (b) meet the psychological needs of followers by creating a supportive environment, and (c) create favorable interpersonal relationships with followers (Thompson, 2005; Yang et al., 2011). According to social learning theory, proactive leaders are charismatic and regarded as role models for subordinates (Crant and Bateman, 2000). Previous studies suggest leaders’ s behavior can inspire and empower followers by role modeling (Caza and Posner, 2019; Ogunfowora et al., 2021). Proactive leaders’ role modeling encourages their followers to emulate their values through learning. Studies suggest that proactive leaders are more likely to identify with and gain commitment from their followers (Lam et al., 2018; Huang et al., 2020). Identification with followers may lead to a value internalization process and improve value similarities among leaders and followers (Marstand et al., 2018). Thus, we hypothesize that:

Hypothesis 4: Leader proactivity is positively related to value congruence.

When people perceive that they share similar values with another person, they evaluate the person’s competency and benevolence (Turban and Jones, 1988). Value similarity can improve communication between leaders and followers (Suazo et al., 2005) and build a trusting relationship between them (Elving, 2005). Thus, when subordinates match the values of the leader, they develop a sense of trust in their leaders. We argue that value congruence can enhance followers’ RBSE. When people experience value congruence with leaders at work, they trust their leaders, which in turn increases their confidence in their decision making in their work (Lau et al., 2007). Trusted employees are offered more opportunities, and they tend to have the confidence to perform activities above and beyond their written in-role job descriptions (Settoon et al., 1996). Furthermore, Byza et al. (2019) suggest that value congruence makes followers experience being appreciated by leaders, which makes followers feel empowered and ultimately facilitates their self-efficacy. In previous studies, Lee et al. (2017) suggest that the consistency of values among followers and ethical leaders can enhance the development of followers’ moral efficacy. Furthermore, value congruence between leaders and followers can increase followers’ FRCC, which refers to the psychological state in which an employee feels a personal obligation to make a constructive change (Morrison and Phelps, 1999). Value similarity can foster trusting relationships between leaders and followers, and trusting relationships can garner employee support for change. According to Albrecht and Travaglione (2003), employees in trusting relationships are willing to take action under conditions of uncertainty or risk. Additionally, social exchange theory describes the sense of obligation to reciprocate in the social exchange process, such as in high-quality relationships characterized by trust or support. This implies that employees feel an obligation to reciprocate with their organization by displaying positive attitudes and behaviors when they believe their leader trusts them (Settoon et al., 1996; Basit, 2017). Erkutlu and Ben Chafra (2016) propose that when individuals have values similar to those of their leaders, they are more inclined to commit to change.

As elaborated above, we expect leader proactivity to trickle down to follower proactivity through the chain mediation of value congruence, RBSE, and FRCC. Based on social learning theory, followers are more inclined to emulate the values of a proactive leader serving as a role model. This develops followers’ RBSE and FRCC and then facilitates followers’ proactive behavior. Based on the discussion above, we propose the following hypothesis:

Hypothesis 5: Value congruence and RBSE play a chain mediating role in the relationship between leader proactivity and follower proactivity.

Hypothesis 6: Value congruence and FRCC play a chain mediating role in the relationship between leader proactivity and follower proactivity.

## Materials and Methods

### Sample

We collected data in three waves from employees of 6 different corporations (two banks, two companies in the high-tech industry, one hotel, and one advertisement company) in Chengdu, Chongqing, and Kunming, which are located in southwestern China. We approached the HR managers of these organizations to seek their cooperation. Finally, six corporations agreed to participate in the survey. Prior to their participation, we informed HR managers of the purpose and process of the study. Participants were recruited through HR managers in their firms. A multi-wave (three-wave) and multisource (supervisor-subordinate dyads) design was used to minimize common method bias [CMB; Podsakoff et al. (2003)].

Questionnaires could be completed in handwritten or email format. In preparing to conduct the surveys, all participants gave their written informed consent prior to the present study. HR supervisors were instructed to recruit leader-follower dyads. We matched the three-phase data through researcher-defined codes. The participants completed the questionnaires on their own time and returned postage-paid return envelopes to the research assistant. Other participants returned the questionnaire by email.

At Time 1, 800 employees provided demographic information and their immediate leaders’ proactivity, and 682 completed the questionnaire, yielding a response rate of 85.17%. At Time 2, participants were asked to report their RBSE, FRCC, and perceived value congruence with their immediate leader, and 602 finished the survey (response rate of 89.05%). At Time 3, we asked subordinates’ immediate leaders to report the proactivity of their subordinates, and we received 122 responses (response rate of 93.84%). After removing missing and unmatched data, we finally obtained 575 valid survey questionnaires. We provided approximately $8 to supervisors and approximately $5 to subordinates as compensation to enhance the quality of the questionnaire data.

### Measures

All survey items were translated from English to Chinese using accepted translation back-translation techniques (Brislin, 1980). All items used a 5-point Likert scale (1 = strongly disagree, 5 = strongly agree). To check the reliability and validity. The initial questionnaire was distributed to 20 professionals (10 professors, five Ph.D. students and five HRM specialists). These professionals suggested modifications to the research measurement tool.

### Dependent and Independent Variables

#### Leader Proactivity

Leader proactivity was measured with a three-item scale from Griffin et al. (2007). An example item is “I initiated better ways of performing my core tasks,” “I made changes to the way your core tasks are done” (Cronbach’s alpha = 0.81).

#### Value Congruence

Value congruence was measured with a three-item scale from Hofmann and Gavin (1998). An example item is “My values are similar to my leader’s values” “I deeply believe in the same ultimate values as my leader does” (Cronbach’s alpha = 0.85).

#### Role Breadth Self-Efficacy

Role breadth self-efficacy was measured using seven items developed by Parker (1998). An example item is “I feel confident designing new procedures in my work,”” I feel confident in representing my work area in meetings with senior management” (Cronbach’s alpha = 0.84).

#### Felt Responsibility for Constructive Change

Felt responsibility for constructive change was measured using five items developed by Morrison and Phelps (1999). An example item is “I feel a personal sense of responsibility to bring about change at work” (Cronbach’s alpha = 0.90).

#### Follower Proactivity

Follower proactivity was measured with a three-item scale from Griffin et al. (2007). An example item is “I initiated better ways of performing my core tasks,” “I feel obligated to try to introduce new procedures where appropriate,” “I made changes to the way your core tasks are done” (Cronbach’s alpha = 0.83).

### Control Variables

Base on previous research (Li et al., 2021), we controlled for the possible confounding effects of employees’ gender, age, education, and tenure.

### Data Analysis Strategy

First, SPSS 24.0 was used to analyze the internal consistency and calculate demographic characteristics and correlations among the study variables. Second, Anderson and Gerbing (1988) two-step approach was utilized to test the proposed chain model. In the first step, confirmatory factor analysis (CFA) was used to test the measurement model using Mplus 7.0 (Muthén and Muthén, 2017) to evaluate the measure’s discriminant validity. In the second step, the hypothesized theoretical model was tested by structural equation modeling (SEM) (see Appendix A). We followed Preacher et al. (2007) suggestions to test the indirect effect and conducted the bootstrap analysis. We first test the effect of leader proactivity on follower proactivity and the effect of leader proactivity on value congruence. Then, we test the mediating role of RBSE and FRCC in the relation of leader proactivity and follower proactivity. We further test the sequential mediation effect of value congruence and RBSE on the relationship between leader proactivity and follower proactivity and the sequential mediation effect of value congruence and FRCC on the relationship between leader proactivity and follower proactivity. Path analysis using maximum likelihood estimation was conducted to calculate the path coefficients of each hypothesis to test the overall structural relationship and influence. Indirect effects were assessed using a 5,000 bootstrap approach and 95% confidence intervals. The significance of the indirect effect is supported when confidence intervals do not include zero.

## Results

### Descriptive Statistics Results

The sample consisted of 310 women and 265 men (gender: *M* = 0.46, *SD* = 0.49). Their age (age: *M* = 3.05, *SD* = 1.16) ranges were 18–20 years (0.9%), 21–25 years (25.7%), 26–30 years (38.1%), 31–35 years (19.3%), 36–40 years (10%), and 41 years or over (6%). Of the sample, 47.88% had completed a bachelor’s degree (education: *M* = 2.66, *SD* = 0.81). Approximately 88.4% of participants had more than 3 years of organizational tenure (tenure: *M* = 2.66, *SD* = 1.27).

### Measurement Model

Before testing our hypotheses, we conducted CFA using Mplus 7.0 to evaluate the factor structure of our measures. As indicated in Table 1, the CFA of the measured variables showed support for the five-factor solution (leader proactivity, value congruence, FRCC, RBSE, and follower proactivity), showing an adequate fit to the data: χ^2^/*df* = 448.67/176 = 2.54, CFI = 0.93, IFI = 0.93, TLI = 0.92, and RMSEA = 0.060. The convergent validity of the studied constructs was measured by composite reliability (CR) and average variance extracted (AVE). According to previous literature, the acceptable value for CR is 0.7 (Bagozzi and Yi, 1988) and that for AVE is 0.5 (Fornell and Larcker, 1981). The convergent validity results, shown in Table 2, reveal a high level of convergent validity. We also calculated the discriminant validity of the study constructs. Hair et al. (2011) suggest that discriminant validity is achieved when the correlation values of each variable are smaller than the square root of the AVE. As shown in Table 3, the discriminant validity value meets the criteria proposed by Hair et al. (2011).

**TABLE 1:** Confirmatory factor analysis.

Measurement models	χ2	*df*	CFI	TLI	IFI	RMESA
Five-factor	448.67	176	0.93	0.92	0.93	0.06
Four-factor (combining leader proactivity and value congruence into one factor)	542.91	178	0.87	0.86	0.87	0.07
Three-factor (combining leader proactivity and value congruence, and RBSE into one factor)	841.35	179	0.70	0.67	0.70	0.10
Two-factor (combining leader proactivity and value congruence, RBSE and FRCC into one factor)	962.78	182	0.56	0.54	0.56	0.13
One-factor (combining all items into one factor)	1512.55	185	0.35	0.32	0.35	0.17

*CFI, comparative fit index; IFI, incremental fit index; TLI, Tucker-Lewis index; RMSEA, root mean square error of approximation.*

**TABLE 2:** Convergent validity.

Constructs	AVE	CR
Leader proactivity	0.71	0.87
Value congruence	0.77	0.90
RBSE	0.62	0.92
FRCC	0.60	0.88
Follower proactivity	0.77	0.90

*AVE, average variance extracted; CR, composite reliability.*

**TABLE 3:** Discriminant validity.

Constructs	1	2	3	4	5
Leader proactivity	0.84				
Value congruence	0.21**	0.87			
RBSE	0.50**	0.22**	0.78		
FRCC	0.50**	0.22**	0.40**	0.77	
Follower proactivity	0.47**	0.12**	0.42**	0.44**	0.87

n = 575; *p < 0.05. **p < 0.01

### Correlation Analysis

The descriptive statistics, correlations, and reliability of the study variables are shown in Table 4. As expected, leader proactivity (*M* = 3.73, *SD* = 0.67) (Time 1) is positively and significantly related to value congruence (*M* = 3.33, *SD* = 1.00) (Time 2) (β = 0.26, *p* < 0.01), RBSE (*M* = 3.35, *SD* = 0.48) (Time 2) (β = 0.50, *p* < 0.01), FRCC (*M* = 3.57, *SD* = 0.61) (Time 2) (β = 0.50, *p* < 0.01), and follower proactivity (*M* = 3.86, *SD* = 0.59) (Time 3) (β = 0.47, *p* < 0.01). Value congruence (Time 2) is positively and significantly related to RBSE (Time 2) (β = 0.22, *p* < 0.01), and FRCC (Time 2) (β = 0.22, *p* < 0.01), and follower proactivity (Time 3) is positively related to RBSE (Time 2) (β = 0.42, *p* < 0.01) and FRCC (Time 2) (β = 0.44, *p* < 0.01).

**TABLE 4:** Results of descriptive statistical analysis.

Variables	*M*	*SD*	1	2	3	4	5	6	7	8	9
1. Gender	0.46	0.49									
2. Age	3.05	1.16	–0.01								
3. Education	2.66	0.81	–0.08	–0.03							
4. Tenure	2.66	1.27	–0.01	0.62**	0.00						
5. Leader proactivity (Time 1)	3.73	0.67	–0.13	–0.00	0.13	–0.00	0.81				
6. Value congruence (Time2)	3.33	1.00	–0.05	0.03	0.01	–0.00	0.21**	0.85			
7. RBSE(Time2)	3.35	0.48	−0.11*	0.06	0.05	0.10*	0.50**	0.22**	0.84		
8. FRCC (Time 2)	3.57	0.61	–0.07	–0.01	0.13**	0.03	0.50**	0.22**	0.40**	0.90	
9. Follower proactivity (Time3)	3.86	0.59	−0.11*	0.03	0.13**	0.01	0.47**	0.12**	0.42**	0.44**	0.83

n = 575; Cronbach’s alpha reliabilities displayed on the diagonal; *p < 0.05. **p < 0.01.

### Common Method Bias Testing

Common method bias (CMB) was assessed by Harman’s single-factor test (Podsakoff et al., 2003). The total variance of a single factor was 23.31% for all five theoretical constructs in the model, below the suggested minimum of 50%. These results suggest that CMB was not a serious concern in the current study.

### Structural Equation Model Results and Hypothesis Tests

**FIGURE 2:**
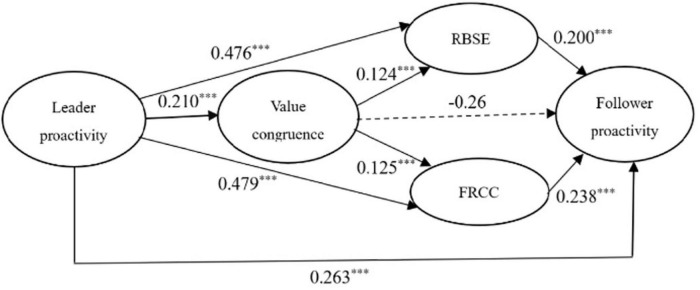
Structural relationships. ^***^< 0.001.

As shown in Figure 2, SEM was used to verify the suitability of the model. The results indicated an acceptable fit: *X^2^/df* = 2.780, CFI = 0.95, TLI = 0.94, SRMR = 0.033, and RMSEA = 0.07(Hu and Bentler, 1999).

The direct and indirect effects are illustrated in Table 5 and Figure 2. To test indirect effects, we followed Edwards and Lambert (2007) and obtained 95% CIs with 5,000 bootstrap estimates using Mplus 7.0. As shown in Table 5, leader proactivity positively and significantly predicted follower proactivity (β = 0.26, *SE* = 0.05, *p* < 0.001) and value congruence (β = 0.21, *SE* = 0.05, *p* < 0.001), RBSE partially mediates between leader proactivity and follower proactivity [indirect effect = 0.08, *SE* = 0.02, 95% CI = (0.051, 0.126)], FRCC partially mediates between leader proactivity and follower proactivity [indirect effect = 0.10, *SE* = 0.03, 95% CI = (0.056, 0.157)], value congruence and RBSE plays a significant chain mediating role in the relationship between leader proactivity and follower proactivity [indirect effect = 0.005, *SE* = 0.002, 95% CI = (0.002, 0.010)], and value congruence and FRCC plays a significant chain mediating role in the relationship between leader proactivity and follower proactivity [indirect effect = 0.005, *SE* = 0.003, 95% CI = (0.002, 0.013)], these results support Hypotheses 1–6.

**TABLE 5:** Summary of hypothesis testing.

	Pathway	Estimate	S.E.	*t*-Value	L.L.	U.L.	Decision
H1	LPB→FPB	0.263	0.050	5.254	0.181	0.346	Supported
H2	LPB→RBSE→FPB	0.084	0.023	3.722	0.051	0.126	Supported
H3	LPB→FRCC→FPB	0.100	0.031	3.285	0.056	0.157	Supported
H4	LPB→VC	0.210	0.059	3.561	0.110	0.303	Supported
H5	LPB→VC→RBSE→FPB	0.005	0.002	2.082	0.002	0.010	Supported
H6	LPB→VC→FRCC→FPB	0.005	0.003	1.836	0.002	0.013	Supported

*LPB, Leader proactivity; FPB, Follower proactivity; VC, Value congruence.*

## Discussion

The present study responds to calls for further research on the outcomes of proactivity (Liu et al., 2019) and develops and empirically tests a theoretical model to understand the trickle-down effects of leader proactivity on follower proactivity. As expected, the results of this study reveal that leaders’ proactivity facilitates followers’ value congruence, which in turn enhances followers’ self-efficacy for carrying out broader work roles (RBSE) and responsibility for constructive change (FRCC) and ultimately promotes followers’ proactivity. This study offers several important theoretical and practical contributions.

First, although scholars have stressed the critical role of employee proactive behavior in organization research (Parker et al., 2006, 2019), little literature has been devoted to understanding leader-level proactivity (Crossley et al., 2013; Rashkovits, 2019). Our research found that leader proactivity positively predicts follower proactivity. Consistent with previous studies, this result indicates that leaders play a vital role in motivating followers’ proactive behavior (Griffin et al., 2010; Den Hartog and Belschak, 2012; Pan et al., 2012; Hu et al., 2018; Weiss et al., 2018; Mostafa and El-Motalib, 2019). Based on social learning theory, this result shows that proactive leaders are attractive to employees, and followers are inclined to emulate the proactive behavior of their leaders. This result enriches the understanding of the effects of leader proactivity (Liu et al., 2019). Because proactive behavior can be risky for subordinates, this result suggests that when leaders act proactively, their subordinates will feel safe to engage in exploring new possibilities and taking initiative. These findings answer Parker and Wu (2014) call to consider how different types of leader behaviors motivate followers’ proactivity.

Second, we found that leader proactivity was a strong motivator for value congruence between leaders and subordinates. Corresponding with the social identification perspective, this result confirms the proposition by Crant and Bateman (2000) that proactive leaders are charismatic and regarded as role models for their followers; that is, followers are inclined to emulate the values displayed in leader proactive behavior. Previous research has considered leaders to be the main antecedents of value congruence. However, research has focused predominantly on leadership without considering leader behavior factors. We suggest that proactive leaders can provide clear guidelines and satisfy followers’ social needs; these behaviors enhance employees’ identification with their leaders and increase employees’ commitment (Lam et al., 2018; Huang et al., 2020). Followers’ identification may lead to a value internalization process and improve value congruence between leaders and followers. In contrast to the existing literature, this result extends proactivity research by focusing on the consequences of leader proactivity.

Third, this study provides a comprehensive model that investigates the mediating mechanism of the relationship between leader proactivity and follower proactivity; that is, leader proactivity can affect follower proactivity through value congruence and RBSE *via* a chain mediation path. Although previous research has begun to investigate the consequences of leader proactivity (Parker et al., 2019), empirical findings pertaining to the effects of leader proactive behavior are poorly understood. This finding of a chain mediation model shows that proactive leaders can promote value congruence and then increase follower RBSE, which in turn motivates follower proactivity. The underlying theoretical logic is that proactive leaders’ role modeling encourages their followers to emulate their values through learning, which can shape and develop follower RBSE and then ultimately motivate followers to engage in proactivity. In accordance with social learning theory, this result broadens the proactive behavior literature by strengthening the understanding of the mechanism linking leader proactivity and follower proactivity. Consistent with the proactive motivation model of Parker et al. (2010), this result reveals that leader proactivity can affect follower proactivity through “can do” motivational states.

Fourth, the results confirm that leader proactivity can affect follower proactivity through value congruence and FRCC *via* a chain mediation path. This finding shows that leader proactivity enhances value similarity between leaders and followers, which encourages followers to identify with their leaders and then makes them feel responsible for being proactive. From a social exchange perspective, proactive leadership will be rewarded with proactivity from subordinates. This finding implies that leader proactivity can motivate follower proactivity through a “reason to” path (Parker et al., 2010). Many studies have been devoted to understanding the mechanisms of leader-related factors and followers’ proactive behavior (Gao et al., 2011; Cai et al., 2019). However, we do not have a sufficient understanding of how followers respond to leader proactivity. The finding of a chain mediating role of value congruence and FRCC in the relationship between leader proactivity and follower proactivity adds value to the organizational behavior literature and extends the prior work on leader-related factors and followers’ proactive behavior.

## Implications for Business Practice

Our findings delivered several crucial suggestions for organizations. First, leaders’ proactive behavior is beneficial for motivating followers’ proactive behavior. Given this result, organizations should provide decision-making discretion, broad information, and job autonomy to promote leaders’ proactivity. Because proactive behavior is risky for both leaders and employees, organizations should also incorporate an inclusive organizational culture that is conducive to personal initiative. In the recruitment and selection process, organizations should also focus on selecting leaders with proactive personalities.

Second, value congruence, RBSE and FRCC serve as important mediators that link leader proactivity and follower proactivity. Because proactive leaders can enhance value congruence between them and followers and then motivate follower proactivity with RBSE (“can do”) and FRCC (“reason to”) motivational states, organizations should strengthen the communication between leaders and employees and train their leaders with video modules and one-on-one coaching to demonstrate the importance of value congruence. Organizations should also provide job design (e.g., Job rotation, job enlargement) and training programs (e.g., skills Training Teamwork Training) for employees to enhance their RBSE and sense of FRCC.

Third, RBSE and FRCC provide motivation for employees’ proactive behavior. In workplaces that require employees to respond in a proactive manner, organizations can use RBSE and FRCC as important selection criteria in the process of recruiting (i.e., highly experienced people with proactive personalities). Organizations should also implement organic high-involvement work designs that include on-the-job training, job autonomy, job rotation, and information sharing to promote employees’ RBSE and FRCC.

## Limitations and Future Research

This study had some limitations that should be addressed. First, although research data were collected longitudinally, no strict causal implications can be identified. For example, the relationships between the studied variables are likely to reciprocally influence each other over time. For example, proactive followers can also evoke their leaders’ proactivity through a bottom-up effect. Future research should attempt to use experimental designs and experience-sampling methodologies to provide stronger causal implications. Second, we adopted the social learning perspective as the underlying mechanism linking leader proactivity and follower proactivity. Leader proactivity can also create resources for followers’ proactivity. Thus, future research could explore other mechanisms (such as resource mechanisms) underlying leader proactivity and follower proactivity. Finally, we only carried out data collection based on six Chinese firms, which may limit the generalizability of our findings. Ideally, these findings should be replicated in Western countries to clarify the generalizability.

## Data Availability Statement

The data analyzed in this study is subject to the following licenses/restrictions: for personal privacy, please contact the author if the data is needed. Requests to access these datasets should be directed to ZC, cui_zilong@hotmail.com.

## Ethics Statement

The studies involving human participants were reviewed and approved by Jilin University of Finance and Economics. The patients/participants provided their written informed consent to participate in this study.

## Author Contributions

ZC contributed to study conception theoretical foundation, model development, and research design. KZ contributed to the literature research. Both the authors contributed to analysis, interpretation of data, and drafting of manuscript.

## Conflict of Interest

The authors declare that the research was conducted in the absence of any commercial or financial relationships that could be construed as a potential conflict of interest.

## Publisher’s Note

All claims expressed in this article are solely those of the authors and do not necessarily represent those of their affiliated organizations, or those of the publisher, the editors and the reviewers. Any product that may be evaluated in this article, or claim that may be made by its manufacturer, is not guaranteed or endorsed by the publisher.

## Appendix A

### Mplus Codes

DATA:

 FILE IS Users\

 VARIABLE:

 NAMES ARE gen edu age ten LPB1 LPB2 LPB3 FPB1 FPB2

 FPB3 FRCC1 FRCC2 FRCC3 FRCC4 FRCC5 VC1 VC2 VC3 LPB

 FPB FRCC VC RBSE6 RBSE7 RBSE RBSE1 RBSE2 RBSE3 RBSE4 RBSE5;

 USEVAR ARE LPB FRCC RBSE VC FPB;

 ANALYSIS:

 TYPE = GENERAL;

 BOOTSTRAP ARE 5000;

 MODEL:

 FPB ON VC (b1);

 FPB ON RBSE (b2);

 FPB ON FRCC (b3);

 FPB ON LPB (cdash);

 VC ON LPB (a1);

 RBSE ON LPB (a2);

 FRCC ON LPB (a3);

 RBSE ON VC (d1);

 FRCC ON VC (d2);

 MODEL CONSTRAINT:

 NEW(a1b1 a2b2 a3b3 a1d1b2 a1d2b3 TOTALIND TOTAL);

 a1b1 = a1*b1;! Specific indirect effect of X on Y via M1 only

 a2b2 = a2*b2;! Specific indirect effect of X on Y via M2 only

 a3b3 = a3*b3;! Specific indirect effect of X on Y via M3 only

 a1d1b2 = a1*d1*b2;! Specific indirect effect of X on Y via M1 and M2

 a1d2b3 = a1*d2*b3;! Specific indirect effect of X on Y via M1 and M3

 TOTALIND = a1b1 + a2b2 + a3b3 + a1d1b2 + a1d2b3;! Total indirect effect of X

 TOTAL = a1b1 + a2b2 + a3b3 + a1d1b2 + a1d2b3 + cdash;! Total effect of X on Y

 OUTPUT:

 STAND CINT(bcbootstrap);
